# Semi‐passive monitoring of game play detects learning curves distinctive to subjective cognitive decline (SCD) and mild cognitive impairment (MCI)/mild‐stage dementia

**DOI:** 10.1002/alz.086869

**Published:** 2025-01-09

**Authors:** Chao‐Yi Wu, Jake A Galler, Nadine A Schwab, Barnaly Rashid, Cathrine Young, Liuqing Yang, Jessica A. Gerber, Hiroko H Dodge, Steven E. Arnold

**Affiliations:** ^1^ Massachusetts General Hospital, Harvard Medical School, Boston, MA USA; ^2^ Abbvie Inc., North Chicago, IL USA

## Abstract

**Background:**

The ability to monitor cognitive trajectories over the course of trials can provide valuable insights into treatment efficacy. However, existing trial methods are limited in monitoring cognition in real‐time and at high frequencies. Gameplay‐based assessments hold promise as complementary cognitive tools. In this study, we investigated the learning curves of gameplay features linked to cognitive impairment.

**Method:**

MIND GamePack is an iOS‐based system that includes a set of four games: Memory Match (Card matching), Word Scramble (Boggle), FreeCell (Solitaire), and Block Drop (Tetris). Sixty participants included 26 older adults with intact cognition, 11 with subjective cognitive decline (SCD) and 23 with mild cognitive impairment (MCI)/mild‐stage dementia. Participants were asked to play each game for at least five minutes/day and five days/week for four months. Latent trajectory models were used to detect learning curves distinctive to persons with SCD and MCI/mild‐stage dementia as compared with normal.

**Result:**

For Memory Match, the latent trajectory model identified two learning curves for card matching accuracy. Participants with MCI/mild‐stage dementia were more likely to belong to a group where their accuracy exhibited a slight improvement but remained consistently below‐average throughout the monitoring period (odds ratio [OR] = 3.17, p<.001). Two learning curves were identified for the self‐chosen game level. Participants with MCI/mild‐stage dementia were more likely to belong to the flat curve group that chose the same game level throughout the monitoring period (OR = 1.98, p = .01). For Word Scramble, two learning curves were identified for word discovery rate and word repetition rate, respectively. Participants with MCI/mild‐stage dementia were more likely to belong to the group that demonstrated a decrease in the word discovery rate and an increase in word repetition rate throughout the monitoring period (OR = 2.91, p = .001; OR = 3.17, p<.001). For FreeCell, two learning curves were identified for the percentage of incorrect moves. Those with SCD and MCI/mild‐stage dementia were more likely to belong to the group that showed a higher percentage of incorrect moves at the beginning but gradually decreased throughout the monitoring period (OR = 2.01‐2.62, p = 0.03‐0.003).

**Conclusion:**

Learning curves of game features associated with memory and language can distinguish cognitive severities. Changes in gameplay learning patterns could serve as additional evidence of potential treatment effects.

